# The use of PET/CT in radiotherapy planning: contribution of deformable registration

**DOI:** 10.3389/fonc.2013.00033

**Published:** 2013-04-12

**Authors:** Ela Delikgoz Soykut, Esat Mahmut Ozsahin, Yildiz Yukselen Guney, Suheyla Aytac Arslan, Ozlem Derinalp Or, Muzaffer Bedri Altundag, Gamze Ugurluer, Pelagia G. Tsoutsou

**Affiliations:** ^1^Radiation Oncology, Dr. Abdurrahman Yurtarslan Ankara Oncology Education and Research HospitalAnkara, Turkey; ^2^Radiation Oncology, Lausanne University HospitalLausanne, Switzerland; ^3^Adana Acibadem HospitalAdana, Turkey

## Introduction

Medical imaging provides information for diagnosis and staging, evaluation of treatment response, and also plays pivotal role in advanced radiotherapy treatment planning. In the era of the innovative conventional and functional imaging techniques, the main goal of radiation therapy, which is to maximize the dose to the target while minimizing the dose to adjacent healthy organs, can be actualized. On the basis of this, accurate delineation has led to the safe decrease of radiotherapy volumes, in terms of resulting in a reduced risk of normal tissue toxicity, and increasing tumor control probability (De Ruysscher et al., 2005; van Der Wel et al., 2005).

Computed tomography (CT) is the primary modality for image-based treatment planning, but conventional anatomic imaging with CT has limited sensitivity to identify distinctly the anatomic borders of the tumor (Nestle et al., 2009). Integration of multimodality imaging data for radiotherapy treatment planning is beneficial and indespensable for perfect delineation (Kessler et al., 1991; Rosenman et al., 1998).

Currently, several studies showed that positron emission tomography–computed tomography (PET/CT) is significantly used for staging for various type of tumor. PET/CT combines biological activity and anatomical information in a single study session, and provides to distinguish viable tumor focus. The use of PET/CT for fused images with planning CT (pCT) may allow adequate tumor visualization (De Ruysscher et al., 2005; van Der Wel et al., 2005). Otherwise, implementation of PET/CT information for radiation therapy planning (RTP) to accurate delineation is under investigation, but is not recommended for a routine procedure. In this paper, we focus on the clinical adoption of PET/CT and the use of PET/CT in RTP.

## The role of PET/CT in target definition

PET/CT is preferred medical imaging modality for detecting atelectasis from intratumor heterogeneity in non-small cell lung cancer, and has higher sensitivity and specificity for identifying lymph node involvement (Nestle et al., 1999). In several planning studies, it was shown that the addition of PET/CT information is associated with smaller size on tumor volume than compared with radiotherapy pCT (Bradley et al., 2004; Hanna et al., 2010; Moller et al., 2011), thus radiation oncologists enable to allow dose escalation with slightly lower doses on organs at risk with promising high curability rates. The problem of target motion is an important topic for lung and other thoracic malignancies. Respiratory motion can cause artifacts that potentially deteriorate the quality of images and the appearance of tumor with resulting misdiagnosis and mislocalization (Nehmeh et al., 2002; Nehmeh and Erdi, 2008). Nowadays, the use of gated PET/CT and 4-dimensional CT acquisition are becoming more popular for this aspect.

The use of FDG-PET has become standard in the management of both non-Hodgkin's and Hodgkin's lymphoma with its high sensitivity for defining disease (Jerusalem et al., 1999; Kostakoglu and Goldsmith, 2000). PET/CT has a well-accepted role on staging, radiation therapy field design and response evaluation in lymphoma patients and may be predict disease outcome. Recent findings showed that staging with PET/CT is superior than CT or MRI. PET/CT influences in involved field radiation therapy field design in Hodgkin's lymphoma and its essential use leads to help to reduce target volumes while protecting geographic miss (Jerusalem et al., 1999; Mikhaeel et al., 2005). Information provided by PET/CT has distinguished between viable tumor and fibrosis in residual mass during active follow-up.

Although FDG-PET has a low sensitivity for detecting involvement of lymph nodes in early stage cervical carcinoma, it is essential to determine the lymphatic spread especially in para-aortic region for locally advanced disease (Dolezelova et al., 2008). In recent years, it was shown that PET/CT affects radiotherapy planning by tailoring field design and customizing the radiation dose (Salem et al., 2011). PET/CT based brachytherapy optimization allows improved tumor volume dose distribution without significantly increasing the dose to the surrounding organs (Lin et al., 2007).

Because of the high back-ground glucose metabolism of normal gray matter structures, PET/CT with amino acid tracers is used for primary brain tumors instead of FDG-PET. In several studies, it was shown that integration of PET/CT may discriminate active tumor and radionecrosis, and may improve delineation skills and may help to evaluate response after completion of therapy (Ogawa et al., 1993).

Also, PET/CT is recommended medical imaging technique in head and neck cancer, with the advantage of defining disease, nodal status and distant metastases. PET/CT is considered a complementary technique to facilitate the delineation skills on gross tumor volume and to identify nodal status of head and neck cancer (Minn et al., 2010; Zygogianni et al., 2012).

PET/CT has a higher specificity for detecting occult metastases and distant nodal involvement in esophageal tumors, by contrast, has a lower sensitivity than compared with the use of combined endoscopic ultrasound guided CT for identifying lymph nodes which are adjacent to tumor (Mujis et al., 2010). In this regard, PET/CT provides improved staging opportunity, determines extensiveness of disease and supports for treatment decision making, but its use for RTP is limited.

The role of PET/CT in rectal cancers is limited and further studies are needed for a clear data. As evidence accumulates that positive predictive value of PET/CT is high for detecting lymph node involvement instead of its lower sensitivity, radiotherapy fields should be encompass that regions, this situation should not be ignored (Kantorova et al., 2003).

## The use of diagnostic PET/CT for image acqusition and registration

The diagnostic PET/CT is acquired with the patient' arms raised above and laid down a curved couch top. Whereas, the radiotherapy simulation CT images are obtained in a procedure with the patient lying on a flat table. For appropriate fusion images, PET/CT should be performed in RTP position to prevent recumbent position related errors. For this purpose, a second acquisition of PET/CT scan is not acceptable because of the over radiation exposure and additional cost. To solve this issue clinicians use the benefits and functions of improved radiotherapy planning systems.

Correct registration is required to strictly identify gross tumor volume. Rigid and deformable image registrations are the most commonly used subtypes of registration, both can be done in our center by using Velocity Advance Image (AI) Software (Velocity Medical Solutions, Version 2.7, Atlanta, GA). Rigid registration is the overlap of the two image data set based on bony structures. But, the potential differences between image data set, such as variations in anatomical positioning, are still continued by rigid fusion. The probability of mismatch between PET/CT and pCT caused by different recumbent position, unintended organ movements and different respiratory phases is still disadvantage of rigid fusion. It is well known that if the diagnostic PET/CT is acquired the patient lying on a flat table like RTP position, the accuracy of rigid registration increases (Nestle et al., 1999). Whereas in clinical use this procedure is not routine. This requirement has led to the development of deformable registration approaches. Deformable image registration ensures to reduce geometric differences between the two image data sets, by estimating the spatial relationship between the volume elements of corresponding structures. Deformable registration can be executed automatically by using “Navigation” tool which is in the new version or by two steps, initially manually alignment for rigid registration, and then choosing “region of interest” area. Thus, a new close to real image is obtained by this algorithm. Deformable registration has been demonstrated to improve the accuracy of diagnostic PET/CT fusion in head and neck patients, but it is recommended to be careful on delineating tumors in neck region (Hwang et al., 2009).

## Threshold segmentation

The most commonly used PET tracer is 18-F fluorodeoxyglucose (18 F-FDG). The standardized uptake value (SUV) is often used for quantitative analysis of PET and shows the biological activity. Due to the knowledge of the highest FDG uptake area in the pre-treatment session has the highest risk for local recurrence after therapy, some authors suggested that improved local control rates can be obtained by intensifying radiation dose to that areas (Abramyuk et al., 2009). This assessment is significantly important because of allowing better ratios of curability by escalating the dose to the active tumor as well as lowering toxicity by reducing the dose to critical organs. However, the problem of this setting is still unknown which SUV threshold is appropriate, further studies investigated to define optimal SUV levels for many cancers (Wang et al., 2012). Identifying the volume of each SUV is not possible manually, so that automatically tools are required for practical use by Velocity AI. The delineated volumes should be checked on the pCT images and parts of overlapping on bony and airy structures should be deleted.

## The role of PET/CT on stereotactic radiosurgery and stereotactic body radiotherapy

Stereotactic radiosurgery/radiotherapy (SRS) and stereotactic body radiotherapy (SBRT) are advanced radiotherapy techniques which have high degrees of precision. Delivery of high doses to target volume, and limiting the dose to the adjacent normal tissues by rapid dose fall-off, the new stereotactic techniques are effective and safe. Currently, this novel technology gained experience in many type of cancer management in a short time interval, and accepted therapy for a wide range of indications. So, perfect delineation skills are required to deliver doses of radiation with high accuracy. In this context, PET/CT takes place for defining tumor volume in stereotactic approaches.

## The impact of PET/CT on interobserver and intraobserver variabilities

Inspite of ongoing developments in diagnostic imaging and advances on radiotherapy treatment planning, radiation oncologists still manually contour the tumor, and this component is significantly related with interobserver and intraobserver contouring variabilities. This well-defined uncertainty is operator-dependent process that influences target volume and modifies the treatment plan. With the use and interpretation of medical imaging techniques, improvement on interobserver and intraobserver agreement is demonstrated in several studies (Mah et al., 2002). PET/CT increases the interobserver agreement on tumor delineation, aids to avoid geographical misses (Steenbakkers et al., 2006; Buijsen et al., 2012). Also by using these automatic tools mentioned above interobserver and intraobserver variabilities for tumor delineation are reduced.

## Assessment of pre-treatment vs. post-treatment PET/CT findings

**Figure 1:**
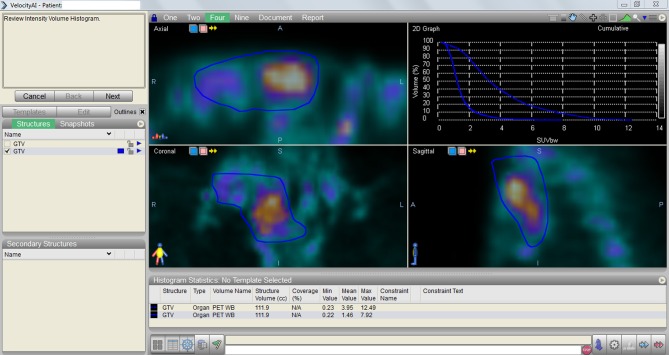
**Displayed the assessment graphic containing the data obtained by the comparison of pre-treatment vs. post-treatment PET/CT findings by using Velocity AI “Navigation” tool**.

PET/CT provides data for evaluation of tumor response for aforementioned types of cancer after completion of therapy. PET/CT is accepted modality for evaluation of treatment response in various types of cancer by means of distinguishing tumor progression, regression or stable disease. We have opportunity to compare the difference between the two images, pre-treatment vs. post-treatment PET/CT, and to obtain an assessment graphic by using Velocity AI “Navigation” tool. This dose-SUV histogram is instructive for clinicians to interpret PET/CT findings and to compare tumor biology (Figure 1).

## Conclusion

Radiation therapy plays a key role in the management of cancer treatment. The main objective of radiotherapy is to achieve improved local control with dose escalation to the tumor while decreasing the probability of side effects by reducing radiation exposure to healthy surrounding organs. Accurate delineation is required for correct definition of gross tumor volume to avoid undertreatment. CT based RTP just includes anatomical information, is insufficient for this aspect, thus advanced imaging techniques have gained critical importance. PET/CT is increasingly being used one of the medical imaging for this purpose that combines the metabolic and anatomic features. PET/CT is accepted modality for diagnosis, staging, and assessment of tumor response in various types of cancer. There are growing data about comparing the use of PET/CT vs. other imaging techniques for RTP, moreover, controversy exists about the appropriate use of PET/CT. Finally, the evaluation of PET/CT images is beneficial for accurate delineation and also a complementary method to determine target volumes in radiotherapy. By employing PET/CT information with the capabilities of Velocity AI as software based image co-registration, threshold segmentation, response assessment can be done practically in radiotherapy planning and follow-up.
